# Relationship Between Fatigue Index and Number of Repetition Maxima with Sub-Maximal Loads in Biceps Curl

**DOI:** 10.2478/hukin-2013-0057

**Published:** 2013-10-08

**Authors:** Ekim Pekünlü, Ozan Atalağ

**Affiliations:** 1 Coaching Education Department, Division of Movement and Training Sciences, School of Physical Education and Sports, Ege University, Bornova, İzmir, TÜRKİYE.; 2 Graduated from Graduate School of Health Sciences, Department of Movement and Training Sciences in Sports, Ege University, Bornova, İzmir, TÜRKİYE.

**Keywords:** resistance exercise, relative load, volitional failure, muscular endurance

## Abstract

The aim of this study was to investigate the relationship between the number of repetition maxima to volitional failure (RM) at 60%, 75%, 90% of 1RM and fatigue index (FI), a determinant of the muscular endurance level. Thirty four resistance trained male participants attended two testing sessions. The first session was conducted to assess 1RM load and RM at 60%, 75% and 90% of 1RM in the supine biceps curl (SBC) exercise. In the second session, a FI test protocol consisting of five sets of SBC with 90 s rest between sets was performed to determine FI values. Each set was performed to volitional failure using a sub-maximal load in the range of 15-20RM. Hypothetical high FI and low FI groups (17 participants with the highest and lowest FI values, respectively) were formed for statistical analyses. ANOVA results revealed that RM at 60%, 75%, 90% of 1RM were not significantly different between FI groups when controlled for mean repetition tempo (p=0.11, p=0.38, p=0.13, respectively). Pearson’s correlation coefficients revealed that no significant relationship was present between FI values and RM at 60%, 75%, 90% of 1RM (p=0.40, p=0.46, p=0.14, respectively). In conclusion, the muscular endurance level of participants defined in terms of FI value was not an indicator of RM in SBC. Therefore, athletes with different muscular endurance levels can use similar percentages of 1RM in biceps curl exercise in their training programs when the aim is to elicit training adaptations related to specific RM zones.

## Introduction

Maximal strength, hypertrophy, muscular endurance and power can be enhanced by properly periodized resistance training (RT) programs (Bird et al., 2005). In order to achieve pre-planned goals in RT programs, several variables should be taken into consideration. Exercise load, number of sets and repetitions, rest intervals, order and choice of exercises are commonly used variables for the prescription of exercise programs.

The relationship between the number of repetitions and intensity, which is defined as the amount of resistance used, is one of the most commonly studied issues in sports science. The number of repetitions maxima to volitional failure (RM) and specified percentages of one repetition maximum (%1RM) are the most common and easiest methods to define the intensity of resistance training exercise (Fleck et al., 2004; Whyte, 2006). A variety of charts and tables are generally used to define the relationship between RM and %1RM. In addition, various prediction equations based on RM are also used to predict the one repetition maximum load (1RM) in resistance training exercises (Ratamess et al., 2011). The common use of these charts, tables and prediction equations are integrated into the development of training plans. Accordingly, specific RM and %1RM zones are used to elicit specific training adaptations (Fleck and Kraemer, 2004; Rippetoe et al., 2006; Whyte, 2006). However, the relationship between RM and %1RM in these charts and equations is based on an unproven assumption of linearity, which can be misleading in practical applications (Fleck and Kraemer, 2004; Whyte, 2006). Studies have demonstrated that RM performed at %1RM can show a high degree of variability (Shimano et al., 2006; Douris et al., 2006; Salvador et al., 2005; Terzis et al., 2008). This variability mainly results from the training experience of athletes, exercise type (single or multi joint), repetition velocity (tempo), type of muscular contraction, muscle mass involved in performed exercise, and neuromuscular control of involved muscles (Sakamoto et al., 2006; Fleck and Kraemer, 2004; Whyte, 2006; Ratamess and American College of Sports Medicine [ACSM], 2011). Another important variable in this context is the local muscular endurance characteristics of active muscles recruited in the performed exercise (Douris et al., 2006; Terzis et al., 2008).

Local muscular endurance is defined as the ability of a muscle or a muscle group to continue contracting against a sub-maximal load in a specific time period or for a specific number of repetitions (Whyte, 2006). Muscular endurance is highly dependent on the muscle fiber type, which is one of the most important determinants of muscle fatigability (Mileva et al., 2009). Slow twitch muscle fibers have higher levels of fatigue resistance than fast twitch muscle fibers (Fleck and Kraemer, 2004; Douris et al., 2006). Great variability exists among individuals as well as among muscle groups in regard to muscle fiber type composition (Karp, 2001). Slow twitch dominant individuals have a tendency to perform a higher number of repetitions at the same %1RM than individuals with a predominance of fast twitch muscle fibers due to their higher endurance levels (Douris et al., 2006; Karp, 2001). It has been also established that local muscular endurance characteristics and, accordingly, resistance exercise performance regarding RM with sub-maximal loads are highly associated with muscular capillary density (Terzis et al., 2008).

Muscular endurance levels should be considered carefully if intensities in RT programs are to be based on %1RM. The use of %1RM instead of RM without considering muscular endurance levels of athletes can cause underestimation or overestimation in the determination of training loads. While underestimation of training loads can lead to insufficient training stimulus, avoiding athletes from reaching specific adaptation levels related to their needs, overestimation can create high risks of injury and/or overtraining. As the genetic predisposition of muscle fiber types is the major factor on which training adaptation depends, specific individual repetition range should be used in resistance training programs so as to gain specific adaptations (Karp, 2001).

Although it has been stated in the literature that a muscle fiber type (Douris et al., 2006) and muscular capillary density (Terzis et al., 2008) are the major factors determining a local muscular endurance level of a muscle or muscle group, it should also be considered that there are many other internal and external variables contributing to the endurance level. Body posture, amount of resistance, contraction type and velocity, muscle mass, muscle length and pulling angle of tendon(s), pennation structure and angle of muscle fibers; cross sectional area, use of energy substrates, contractile function, metabolic capacity, neuromuscular activation patterns, sensory-motor coordination mechanism, muscle membrane excitability of recruited muscles constitute the internal variables (Mileva et al., 2009; Lariviere et al., 2006; Kent-Braun et al., 2002; Battineli, 2007), and training status, nutritional and environmental conditions, and living habits are some of the external variables (Ma et al., 2011). Contribution levels of these variables to endurance characteristics of a muscle or muscle group are very hard to determine due to nonlinear complex interactions among these variables. Therefore, it is reasonable to state that explaining the relationship between endurance levels and RMs in a resistance training exercise with a reductionistic approach, solely based on fiber type distribution or capillary density, is an unrealistic goal. Quantification of a single variable, which can be regarded as the representative of integrated effects of above mentioned variables, could be a holistic approach in the determination of muscular endurance levels. In this context, assessment of muscular endurance levels of athletes with a practical testing method is of great importance both in sport science studies and in designing individualized RT programs.

A practical testing procedure that could be used as a means of roughly estimating the fiber type distribution of recruited muscle group(s) in a resistance exercise has been suggested in the literature. This procedure is based on the RM performed at 80% of 1RM. According to this procedure, individuals who perform 12 or more repetitions in a specified resistance exercise are regarded as slow-twitch fiber dominant individuals, whereas individuals who perform 7 or less repetitions are regarded as fast-twitch fiber dominant individuals. Accordingly, individuals performing 7–12 repetitions at this relative load are regarded as participants having equal proportions of slow and fast twitch muscle fibers. However, it is stated that this is not a scientifically proven testing procedure as the relationship between RMs and muscle fiber type distribution has not been investigated directly (via muscle biopsy method) for this procedure (Karp, 2001). It is also of importance to note that a testing procedure that has the potential to assess the actual muscular endurance level of individuals, rather than fiber type distribution, would be a much more valuable tool in practical sense. Therefore, an adapted version of a fatigue index testing procedure available in the literature (Surakka et al., 2005; Glaister et al., 2008) was used in the assessment of endurance levels of participants in this study.

Recovery is defined as the process of attaining a baseline homoeostasis level after having responded to a training stimulus (Lambert et al., 2005). Recovery ability from local muscular fatigue can be used as an indicator of muscular endurance levels of athletes (Glaister et al., 2008). Muscular fatigability levels and recovery capacities are basic components of athletic performance related to endurance. These components show high inter-individual differences due to genetic factors (e.g. muscle fiber types, metabolism) as well as specific adaptations induced as a response to training (training type, intensity, duration etc.) and various external stimuli (nutrition, environmental factors, living habits etc.). In literature, various kinds of scales, norms and equations under the names of fatigue level, fatigue percentage, fatigue resistance, fatigue index (FI) are used in the quantification of local muscular fatigue (Kumar et al., 2004; Dipla et al., 2009) as well as recovery and endurance levels (Place et al., 2009; Sinacore et al., 1994; Lariviere et al., 2006). Recovery levels of athletes can be assessed according to data obtained from these fatigue measures calculated by the use of specific variables related to the performed physical exercises. Decrement in RM, time under tension (TUT) and impulse (combined effect of RM and TUT) across successive sets can be used as a measure of fatigue and recovery levels in the context of RT.

The purpose of this study was to investigate the relationship between the number of repetition maxima at specified percentages of 1RM and fatigue index. Fatigue index values of participants were calculated according to percentage reduction in the generated relative impulse (RI) measures across 5 sets of bilateral supine biceps curl (SBC) exercise.

High and low FI values in this study represented low and high endurance levels of participants, respectively. The first hypothesis of this study was that a statistically significant negative correlation would be found between FI values and performed RMs at specified percentages of 1RM. After the completion of data collection process, two groups with equal sample sizes (n=17) were formed. One of these groups (low FI group) consisted of 17 participants with the lowest FI values of the whole sample, whereas the other group (high FI group) consisted of 17 participants with the highest FI values. Depending on above mentioned distribution of participants, our second hypothesis was that low FI group would perform significantly greater number of RM at specified percentages of 1RM compared to high FI group.

Besides, a suggestion of a practical test procedure that could support coaches and personal trainers to develop individualized resistance training program prescriptions with effective training loads to elicit specific adaptations constituted an important goal of this study. This procedure is based on assessment of endurance levels of individuals

## Material and Methods

### Participants

Thirty four resistance trained males, most of whom were physical education and sports students in Ege University (age: 22.9 ± 2.7 years, body mass: 79.0 ± 9.1 kg, body height: 181.3 ± 6.9 cm) volunteered to participate in this study. Participants had minimal two years of resistance training experience and were familiar with biceps curl exercises. They were also performing routine resistance training exercises at least 3–4 hours/week and all were free from any upper body injuries at the time of the study for at least one year. All testing sessions were conducted in the fitness center of School of Physical Education and Sports in Ege University.

Participants were instructed not to eat two hours before testing sessions. They were also asked to refrain from drinking alcohol, caffeinated beverages or participating in vigorous physical activity 24 hours before each testing session. Procedures, aims and risks of the study were explained in detail to all participants and they signed an informed written consent form. Approval was granted from Medical Ethics Committee of the Medical Faculty of Ege University (approval number: 12-6/51), in accordance with the Declaration of Helsinki.

### Procedures

In the first session of the study, after the completion of a general warm-up (6-minute running and dynamic stretching), participants performed a specific warm-up procedure on cable machine (ES 180 Cable Crossover Station, ESJIM Ltd., Eskişehir, Turkey) which was also used in the testing protocol. Specific warm-up procedures consisted of three sets of sub-maximal biceps curls with progressively increasing loads (8, 4 and 2 repetitions at 60%, 75% and 90% of their self-estimated 1RMs) separated by one-minute rest intervals.

In this study, a successive eccentric and concentric phase of SBC completed with a good form was accepted as one repetition. Therefore, first repetition of each set was started with an eccentric phase. In the starting position of SBC, legs were straight and kept together, heels were touching each other, elbow joints of the participants were fully flexed, upper arms of participants (triceps part of the arms) were in touch with the floor and as close to their bodies as possible. This position was maintained during each repetition performed in RM tests. Any violation of testing position was not allowed and repetitions not performed in this manner were not regarded as a completed repetition.

Dynamic 1RMs of participants were assessed within 4 trials with a sensitivity of 2 kg. Trials were separated by 3-minute rest intervals. Each participant was asked to estimate their 1RM load considering the load that they had used in the warm up repetitions at 90% of self-estimated 1RM. 1RM trials were initiated with an eccentric phase. Initial load in the first trial was adjusted according to their estimations and they were asked to perform only 1 repetition with this load. With the help of two researchers, each participant held the loaded cable machine straight bar with fully flexed elbow joints. They were instructed to lower the bar in a controlled manner and try to complete the repetition exerting maximal effort after they felt the slight touch of the bar on their quadriceps muscles. Depending on the success or failure in the first trial, the load was either increased or decreased by 2 kg. After each trial participants were asked to comment on the magnitude of the load in order to make proper load adjustments (in some cases, increment and decrement of 4 kg was performed, accordingly). This process was repeated during 3 trials. In the fourth trial, participants used the highest load that they failed in the previous trials. If they succeeded, this load was accepted as their 1RMs. In case of failure, the highest load at which they had succeeded in the previous trial was taken into consideration. Five minutes after the completion of 1RM assessment protocol, the participants performed one set of maximum repetitions to volitional failure at 90%, 75% and 60% of their 1RMs with an 8 and 10-minute rest interval, respectively.

Repetition velocity (tempo) is an important factor in the assessment of RM at %1RM, as RM performed with a sub-maximal load with high velocity are greater than RM performed with low velocity (Sakamoto and Sinclair, 2006; Hatfield et al., 2006; LaChance et al., 1994). Therefore, repetitions were performed with a 3-second (2-second eccentric and 1-second concentric phase) constant repetition tempo in this study. Duration of each repetition was loudly counted second by second by one of the researchers (the same researcher in each test and for each participant) during sets so as to be heard clearly by the participants. No pause was allowed between repetitions. Participants lowered the bar until they felt the slight touch of the bar on their quadriceps in the eccentric phase. The concentric phase was performed with full elbow flexion. Strong verbal encouragement was given to each participant by the same researcher throughout the testing procedure. Participants were familiarized with the test tempo using a very light load before the actual RM tests. TUT was recorded during each RM test with a handheld stopwatch which was also used for tempo adjustment. The last repetition in each test in which participants were not able to complete the whole concentric phase but were able to lift the bar and hold it at least one second in the position that the forearms of the participants were nearly perpendicular to the floor was counted as a half repetition.

In the second session of the study, which was conducted at least 48–72 hours after the completion of the first session, an FI test was performed. This test consisted of five sets of SBC separated by 90-second rest intervals. Calculated FI values based on the results of this test were regarded as the local muscular endurance levels of participants. RM and TUTs were recorded during each set in the FI testing protocol. In this protocol, an individualized test load with which the participants were able to complete a minimum of 15 and a maximum of 20 repetitions was used. Relative loads used in resistance training exercises are commonly expressed either as %1RM or RM completed with good form (Bird et al., 2005; Fleck and Kraemer, 2004; Whyte, 2006). RM performed at a specified percentage of 1RM in a resistance exercise show high inter-individual differences (Shimano et al., 2006; Fleck and Kraemer, 2004; Douris et al., 2006; Salvador et al., 2005). Therefore, testing load in FI test was selected based on RM rather than %1RM in this study. In the second session, warm-up protocols, testing position, movement tempo and verbal encouragement were all the same as in the first session.

Since impulse (force × time) is the major source of fatigue during resistance exercises (Zatsiorsky et al., 2006), this variable was taken into consideration in the calculation of FI. Similarly, impulse values obtained during a 30-second isometric leg extension/flexion exercise were used in the calculation of FI values in the study of Surakka et al. (2005). The amount of force generated by the active muscles continuously changes during isoinertial resistance training exercises due to changes in moment arms and length of muscles (Fleck and Kraemer, 2004; Zatsiorsky and Kraemer, 2006). Hence, impulse generated during a SBC set cannot be calculated by simply multiplying a constant force value (e.g. weight of load lifted) by TUT. However, in this study, it was assumed that no inter-individual difference was present in the changing pattern of generated force across the entire ROM and the generated force was directly proportional to the external load. Depending on these two assumptions, constant weight of the FI test load was used in the calculation of impulse value instead of the actual changing force. Absolute impulse measures were normalized to the relative 1RM calculated by allometric scaling (Jaric, 2003) in order to eliminate the effects of inter-individual differences in 1RM and body mass. RI was calculated with the following equation:

Relative Impulse=(Absolute Impulse)/[Allometric 1RM in SBC]=(9.81×FI test load×TUT)/[1RM×(participant's mass)−0.67]

In the above equation  1RM was defined as the weight of the maximum load lifted in the 1RM test. The weight of the 1RM load and the FI test load were calculated by multiplying the related mass measure by the gravitational constant (9.81 m.s**^−2^**).

FI values of participants were calculated with the following equation adapted from the reliability studies of Surakka et al. (2005) and Glaister et al. (2008):

$$\text{Fatigue}\;\text{Index}=1-\left[\left({\text{RI}}_{2.\text{Set}}+{\text{RI}}_{3.\text{Set}}+{\text{RI}}_{4.\text{Set}}+{\text{RI}}_{5.\text{Set}}\right)/\left(4\times {\text{RI}}_{1.\text{Set}}\right)\right]$$

### Statistical Analyses

Data gathered from this study were analyzed using the IBM^®^ SPSS^®^ version 20 software. Descriptive statistics were expressed as group mean ± standard deviation. A Shapiro Wilk test was performed, and histograms with a normal curve were checked to test the normality assumption of related data. RM performed at 60%, 75%, 90% of 1RM in SBC were the dependent variables (DVs), and the FI groups were the independent variables of this study. Presence of any possible significant correlation between FI and RI_1.Set_ and any possible significant difference in RI_1.Set_ between FI groups in the statistical analyses of this study would indicate that calculated high and low FI values did not result from actual endurance levels of participants, but just from the numerical greatness or smallness of the “RI_1.Set_” variable in the FI equation (denominator of the equation). Therefore, possible relationship between FI and RI_1.Set_, and the significance of the difference in RI_1.Set_ between FI groups were checked by the Pearson’s correlation coefficient and unpaired Student’s t-test, respectively, in order to ensure that the research results were unbiased. Although three-second repetition tempo was given to participants during SBC performance, this tempo unintentionally slowed down, as expected, at the last stages of each set due to fatigue. These unintentional slow repetitions increased the mean repetition tempo (MRT) in performed sets, causing a rise in inter-individual differences in MRT. As the repetition tempo is an important factor in the assessment of RM at specific percentage of 1RM (Sakamoto and Sinclair, 2006; Hatfield et al., 2006; LaChance and Hortobagyi, 1994), this factor was considered to be a possible covariate that could affect DVs in this study. An unpaired Student’s t-test was performed to analyze whether FI groups differed in terms of MRT. After the verification of the homogeneity of regression slope assumption, oneway ANOVA for each DV was conducted to determine the effects of muscular endurance level (FI) on each DV when controlling for MRT. Significance levels of the relationship between FI and RM at 60%, 75%, 90% of 1RM for the whole sample were assessed by the Pearson’s moment correlation coefficient. The level of statistical significance was set at p≤0.05.

## Results

No significant difference was found in physical characteristics between FI groups (Table 1).

No significant relationship was found between FI values and RI_1.Set_ in each FI group and in the whole sample [r(17)=0.37, p=0.14 for low FI group; r(17)=0.03, p=0.92 for high FI group; r(34)=0.26, p=0.15 for the whole sample]. The mean RI_1.Set_ of low FI group was not significantly different from high FI group, t(32)=−0.88, p=0.38, d=−0.30. According to these results, it was concluded that inter-individual differences in the RI_1.Set_ data of participants had no significant negative effects on the outcomes of this study, as well as on the FI values.

MRT of low FI group was not significantly different from high FI group at 60%, 75% and 90% of 1RM [t(32)= −0.30, p=0.76, d=0.10; t(32)= −0.57, p=0.58, d=0.20 and t(32)= −0.39, p=0.70, d=0.13, respectively).

The main effect of FI group (high and low FI groups) on the RM at 60%, 75%, 90% of 1RM was not significant, meaning that adjusted mean RM at 60%, 75%, 90% of 1RM was not significantly different between FI groups (Table 2).

When the whole sample was considered, there was no significant relationship between FI and RM at 60%, 75%, 90% of 1RM [r(34)= −0.15, p=0.40; r(34)=0.13, p=0.46; r(34)=0.26, p=0.14, respectively] showing that muscular endurance levels of participants defined in terms of FI values were not an indicator of RM performed at 60%, 75%, 90% of 1RM in SBC exercise. Also no correlation was found between allometric 1RM of participants and RM performed at 60%, 75%, 90% of 1RM [r(34)=0.38, p=0.16; r(34)=0.03, p=0.85; r(34)= −0.19, p=0.28, respectively].

## Discussion

The main result of this study showed that no significant difference was present in the RM at 60%, 75%, 90% of 1RM between high and low FI groups. In addition, it was found that there was no relationship between FI values and RM performed at these specified percentage of 1RM. These results did not confirm the hypotheses of this study. Doures et al. (2006) found a significant negative correlation between RM performed in leg extension exercise at the load of 70% of 1RM and percentage of fast twitch muscle fibers calculated by the previously developed regression equation. This negative correlation in the study of Doures et al. (2006) could be interpreted as participants having high percentage of muscle fibers with low endurance characteristics-fast twitch fibers-performed less RM.

However, this interpretation is only valid provided that calculated muscle fiber distribution in their study was the representative of the muscle fiber distribution of the whole quadriceps muscle group. When the FI values in our study were assumed to be a function of fiber type distribution of biceps muscles, this result contradicted the findings of our study in the context of endurance characteristics, as no correlation was found between RM performed at 75% of 1RM and endurance levels of participants.

This contradiction could have resulted from highly different designs of the studies in terms of major testing variables. Although similar relative loads were used in both studies, Doures et al. (2006) tested a relatively large lower body muscle group with unilateral isokinetic contractions in their study, whereas in our study a relatively small upper body muscle group was tested with bilateral dynamic (eccentric and concentric) contractions. Acute recovery potentials of large muscle groups (especially in multijoint exercises) tend to be higher than small muscle groups (Willardson et al., 2005; 2006). Therefore, it is highly probable that endurance levels (FI values) of small and large muscle groups differ. This could be one of the main reasons of contradicting results.

Terzis et al. (2008) investigated the relationship between fiber type distributions of vastus lateralis muscle together with capillary density and RM performed at 70% and 85% of 1RM in a leg press exercise. In contrary to the results of the study of Doures et al. (2006), no relationship was found between RM performed at specified percentages of 1RM and fiber type composition of vastus lateralis muscle in their study. Provided the fiber type composition in their study was a determinant of endurance level, this result was consistent with our findings, since no significant relationship was found between RM performed at specified percentages of 1RM and FI, the determinant of endurance level, in our study. In contrary, Terzis et al. (2008) found a significant positive correlation between RM performed at 70% of 1RM and vastus lateralis capillary density. Also a subgroup consisting of six participants with the highest capillary density performed more repetitions at 70% of 1RM than the six participants with the lowest capillary density. Terzis et al. (2008) stated that local muscular endurance was expressed as capillary density in the investigated muscle in their study. Therefore, it could be reasonable to state that their results contradicted our findings since no correlation was found between RM and FI, which was the local muscular endurance level determinant in our study. In addition, absence of a significant difference in RM between different FI groups in our study was the other issue indicating this contradiction which could have resulted from highly different designs of the studies in terms of major testing variables. Firstly, the most profound difference was in the type of exercises. Leg press used in the study of Terzis et al. (2008) is a multi-joint exercise in which several large muscle groups of the lower body are activated, whereas we used a single joint exercise relying on the activation of a relatively small muscle group in the upper body. Secondly, relative loads used in the studies were different. Thirdly, even if a standard repetition tempo was present in the study of Terzis et al. (2008), they did not mention whether there were any inter-individual differences in mean repetition tempo possibly resulting from fatigue occurred at the later stages of the RM sets. Exhaustion moment in a RM set is a function of generated impulse (Zatsiorsky and Kraemer, 2006). Therefore, in the performed set, time under tension is a profound variable that should be taken into consideration. It is reasonable to question whether the results of the study of Terzis et al. (2008) include biases, since the mean repetition tempo, possible covariate that could affect the study results, was not taken into consideration.

The contradictions with regard to consistency aspects of the above mentioned study results were probably due to high differences in study designs and due to the reductionistic approach used in these studies. Doures et al. (2006) conducted their study using a single joint exercise, leg extension, in which quadriceps muscles were recruited as an agonist muscle group. However, Terzis et al. (2008) used a multi joint exercise, leg press, in which quadriceps muscles were recruited as an agonist muscle group together with synergist muscles (gluteus maximus, adductor magnus, soleus). Another difference was between the methods used in the estimation of fiber type distribution. Doures et al. (2006) used a regression equation, whereas Terzis et al. (2008) used the direct method, muscle biopsy. Genders and physical activity levels of the study samples were different as well. Untrained females participated in the study of Doures et al. (2006). In contrary, physically active male participants attended the study of Terzis et al. (2008). These differences could have led to contradicting results. Relying solely on the fiber type distribution or the capillary density, which could be regarded as a reductionistic approach, in the interpretation of study results could be misleading. It was shown that fiber type distribution of the vastus lateralis muscle was different in different depths of the muscle, meaning that the fiber type distribution estimated depending on muscle biopsy samples taken from a single muscle depth cannot be a reliable representative of the whole muscle fiber distribution (Lexell et al., 1983). For example, it has been reported that vastus lateralis and vastus medialis muscles have higher percentages of slow-twitch fibers than rectus femoris muscle in the quadriceps muscle group (Hu et al., 2006). Therefore, biopsies should be taken from at least three different muscle depths of a particular muscle in a muscle group in order to accurately estimate the fiber type distribution of the whole muscle (Lexell et al., 1983). According to this protocol, twelve different biopsy samples are necessary for an accurate estimation when the quadriceps muscle is investigated. However, just a single biopsy sample is taken from a single muscle depth of a single muscle located in the investigated muscle group in studies including biopsy sample analyses. Results of such studies are interpreted based on an unproven assumption that a single biopsy sample taken from the investigated muscle represents the fiber type distribution of the whole muscle or muscle group. Therefore, it should be considered that biased study results are highly probable in such studies.

In another study, it was found that capillary density of a muscle tissue sample taken from a single muscle depth was not a satisfactory representative of whole muscle capillary density in some participants (Dwyer et al., 1999). Especially when the studies are conducted on a large muscle group consisting of several muscles, like in the studies of Terzis et al. (2008) and Doures et al. (2006), biased study results are highly probable in the case that fiber type distribution and capillary density of the whole muscle is estimated depending on biopsy samples taken from a single muscle and single muscle depth. Therefore, defining local muscular endurance as a function of just muscle fiber distribution and capillary density that are estimated in a way mentioned above constitutes a reductionistic approach and is prone to be misleading. Accordingly, quantification of the integrated effects of whole biological and mechanical variables related to local muscular endurance with a practical test procedure, regardless of determining to what degree each variable contributes to the endurance level, has a paramount importance in the context of reliability of study results. This holistic approach certainly provides less speculative knowledge for the sports science literature. In this context, using a single variable (FI) which can be regarded as the representative of total integrated effects of endurance-related characteristics could be identified as a holistic approach in this study.

The result of our study, in the context of RM performed at 60% of 1RM, was inconsistent with the results of the study of Shimano et al. (2006). More repetitions, 19.0 ± 2.9 vs. 15.7 ± 1.9, were performed in their study compared with ours. This inconsistency was probably due to the differences in sample size and repetition tempo as well as possible differences in testing posture and training status of participants. Research data were obtained from a limited number of trained participants (n=8) in the study of Shimano et al. (2006), however, our study was conducted on a greater number of trained participants (n=34). They also used a volitional repetition velocity in their study, which might result in inter-individual differences, whereas a constant repetition tempo was used in our study to avoid biased results as the control of repetition tempo is a crucial factor in studies investigating RM performed at %1RM as well as other resistance training studies (Sakamoto and Sinclair, 2006; Hatfield et al., 2006; LaChance and Hortobagyi, 1994). Participants’ anatomical position during resistance exercise was another crucial factor affecting related exercise performance (Toigo et al., 2006). As the anatomical position related to the performed biceps curl type was not described in the study of Shimano et al. (2006), we could only mention the possibility of posture difference as a factor that might cause inconsistent results in RM. Although large differences were present in performed RM between two studies at 60% of 1RM, very similar results were found in terms of RM at 90% of 1RM. In their and in our study, 4.4 ± 1.9 and 4.7 ± 1.5 repetitions were performed at 90% of 1RM, respectively. According to these results, we could conclude that RM performed at relatively high percentages of 1RM were not affected by the study design factors, mentioned above, as much as RM affected at relatively low percentages of 1RM.

There are limited data related to RM at 75% of 1RM in biceps curl exercise (Ratamess and American College of Sports Medicine [ACSM], 2011). In the study of Shimano et al. (2006) and in the publication of ACSM, performances of 9.1 ± 2.8 and 11.4 ± (no standard deviation was indicated) repetitions at 80% of 1RM were reported, respectively. In the study of Iglesias et al. (2010), 8.8 ± 3.0 repetitions were performed at 70% of 1RM in unilateral preacher curl. These results apparently show how the basic study design factors affect the study outcomes. RM performed with a lighter load are supposed to be more than RM performed with a heavier load, but this was not the case according to results mentioned above. Therefore, interpretation of our findings at 75% of 1RM (10.7 ± 1.7) was highly difficult, according to these results. However, when our findings were evaluated in the context of presented %1RM-RM charts in the publications of National Strength and Conditioning Association (NSCA) (Baechle et al., 2008) and ACSM (Ratamess and American College of Sports Medicine [ACSM], 2011) (4 repetitions at 90% of 1RM and 10 repetitions at 75% of 1RM) our findings were highly consistent (4.7 ± 1.5 and 10.7 ± 1.7 repetitions, respectively). When the RM at 60% was considered, it was hard to mention a consistency since 20 repetitions were reported to be possible according to NSCA (Earle et al., 2004), whereas 15.7 ± 1.9 repetitions were performed in our study. So, it was convenient to conclude that RM performed at medium and heavy loads (75–90% of 1RM) in SBC was consistent with the %1RM-RM charts of ACSM (Ratamess and American College of Sports Medicine [ACSM], 2011) and NSCA (Earle et al., 2004), however, as the load got lighter, this consistency seemed to disappear. Therefore, coaches and athletes should be cautious, especially when determining relatively light training loads according to %1RMRM charts.

It should be considered that the level of replicability of a scientific study design determines its quality. From this point of view, the design of studies cited in the discussion of this manuscript had some shortcomings. Detailed explanations related to extremely important variables, which have direct effects on the study results, were not included in the manuscripts (exact type of the exercise [traditional biceps curl, preacher curl, supine biceps curl etc.], exact body posture, equipment used, features of the equipment used). In addition, some variables, such as repetition tempo and body posture, lacked precise instructions. Therefore, it is hard to interpret and explain actual sources of the contradictions and consistencies in the findings between those studies and ours. Accordingly, it is better to use our study design in further similar studies to obtain reliable results, since it is very clear and simple, as well as includes detailed explanation what makes it easy to replicate.

In conclusion, no relationship was found between FI and RM at %1RM, as well as no difference was detected in RM at 60%, 75%, 90% of 1RM between high and low FI groups. These findings contradicted our research hypotheses. Although, we statistically showed that RI_1.set_ had no significant negative effects on the results of our study using correlation analysis and the unpaired Student’s t-test, this does not mean that each participant had exactly the same RI_1.set_ measure in their FI tests. Still, inter-individual differences existed. It is impossible to identify whether these differences led to biased study results. It should be considered that more reliable study results can be obtained with a study design including a narrower range of RI_1.Set_ and, accordingly, a relationship between FI and RM at 60%, 75%, 90% of 1RM can be detected, if there is any. Also the possibility of non-existence of such a relationship in reality between muscular endurance levels and RM at %1RM in relatively small muscle groups (e.g. biceps brachii in this study) might be one of the explanations for the above mentioned results, since acute recovery potentials of large muscle groups tend to be higher than small muscle groups (Willardson and Burkett, 2005; 2006). This higher recovery potential of large muscles could put forward a more pronounced relationship between RM and FI compared with small muscles. However, more research is needed in this area to make such an interpretation. Therefore, well-structured similar studies should be conducted on small and large muscle groups, as well as on single and multi-joint exercises, to clarify this issue.

In case of the biceps curl exercise, prescribing different exercise loads in terms of %1RM related to specific training zones for athletes with different endurance levels is not necessary, as no difference was found in RM performed at %1RM between FI groups in this study. This issue is also valid for athletes with different relative strength levels. In addition, %1RM-RM charts presented in the publications of NSCA (Earle et al., 2004) and ACSM (Ratamess and American College of Sports Medicine [ACSM], 2011) can be used for a rough estimation of training loads in terms of RM corresponding to medium and high percentages of 1RM (≥75%). However, in case of relatively low percentages of 1RM (≤60%), underestimation of training loads in terms of RM may occur. Therefore, the use of these charts for the prescription of resistance exercise programs including relatively light loads should be restricted.

Defining suitable training loads to elicit specific adaptations in athletes related to their needs is one of the most important issues to be considered in resistance training program prescriptions. However, individual differences in muscular endurance levels of athletes were frequently overlooked. This study attempted to suggest a practical testing procedure that can be used in the assessment of muscular endurance levels of participants in SBC and tested this procedure regarding RM at specified percentages of 1RM. Even if no significant result was achieved at the end of this study, the holistic approach used in this study could be an initiator of similar studies that will be conducted on different muscle groups and different exercises. Accordingly, the use of a simple FI test protocol not only provides a suitable research method in order to assess inter-individual differences between participants regarding muscular endurance characteristics in studies, but also supports coaches and personal trainers to develop individualized resistance training program prescriptions with effective training loads to elicit specific adaptations.

## Figures and Tables

**Table 1:** Physical characteristics of the participants in low FI group (n=17) and high FI group (n=17)

	Group	Mean	SD	Min	Max	t	p	ES
**^1^** Age (y)	Low FI	22.9	2.3	19.1	27.0	−0.13	0.90	−0.04
	High FI	23.0	3.0	18.8	32.2			
**^2^** Resistance Training Age (y)	Low FI	4.6	1.7	2.0	9.0	1.01	0.32	0.35
	High FI	3.9	2.3	2.0	10.0			
Body Height (cm)	Low FI	180.4	8.0	170.0	198.0	−0.81	0.43	−0.28
	High FI	182.3	5.7	173.0	195.0			
Body Mass (kg)	Low FI	79.2	8.1	67.0	93.0	0.14	0.89	0.05
	High FI	78.8	10.1	59.1	102.0			
1RM (kg)	Low FI	36.8	5.7	28.2	51.0	0.27	0.79	0.09
	High FI	36.4	3.9	26.4	40.0			
**^3^** R-Strength (N.kg ^−0.67^)	Low FI	19.3	2.6	16.3	25.4	0.13	0.89	0.04
	High FI	19.2	1.9	16.7	22.8			

y=year; N=Newton; Min=Minimum; Max=Maximum; SD= Standard Deviation; ES=Effect Size (Cohen’s d);

1 Time passed from birth date to the testing date;

2 Time passed since the first participation in complementary resistance training for the specific sport branch;

3 Relative strength according to allometric scaling

**Table 2:** Original and adjusted means of performed RM at 60, 75, 90% of 1RM after controlling for MRTs

			Original Data		Adjusted Data				

	Group	N	Mean	SD	Mean	SE	F	p	**η_p_^2^**
RM at 60% 1RM	Low FI	17	16.3	1.8	16.2	0.44	2.78	0.11	0.08
	High FI	17	15.2	1.9	15.2	0.44			
	Total	34	15.7	1.9	-	-			
RM at 75% 1RM	Low FI	17	10.5	1.7	10.4	0.41	0.79	0.38	0.03
	High FI	17	10.9	1.8	10.9	0.41			
	Total	34	10.7	1.7	-	-			
RM at 90% 1RM	Low FI	17	4.44	1.56	4.39	0.31	2.38	0.13	0.07
	High FI	17	5.00	1.38	5.05	0.31			
	Total	34	4.72	1.48	-	-			

RM=Repetition Maxima; FI=Fatigue Index; MRT=Mean Repetition Tempo; SE=Standard Error; SD=Standard Deviation
